# The Dynamic Behaviour of Multi-Phase Flow on a Polymeric Surface with Various Hydrophobicity and Electric Field Strength

**DOI:** 10.3390/polym14040750

**Published:** 2022-02-15

**Authors:** Qi Li, Rui Liu, Li Li, Xiaofan Song, Yifan Wang, Xingliang Jiang

**Affiliations:** 1 State Key Laboratory of Power Transmission Equipment and Systerm Security and New Technology, College of Electrical Engineering, Chongqing University, Chongqing 400044, China; 201911021010@cqu.edu.cn (R.L.); profjiang2018@gmail.com (X.J.); 2 Electric Power Research Institute of Guangdong Power Grid Co., Ltd., Guangzhou 510080, China; liligz@163.com (L.L.); haomafan2006@126.com (Y.W.); 3 State Grid Henan Economic Research Institute, Zhengzhou 450052, China; sxf810118@sina.com

**Keywords:** droplet vibration, high voltage insulator, polymeric surface, corona discharge, arcing, creepage distance

## Abstract

The dynamic behaviour of rain droplets on the insulator surface is a key measure to its reliability and performance. This is due to the fact that the presence and motion of rain droplets cause intensive discharge activities, such as corona and low current arcing, which accelerate the ageing process and flashovers. This article aims to investigate and characterize the movement of a rain droplet placed on an inclined insulator surface subject to an intensive electric field. The rain droplets’ movement on hydrophobic surfaces in the absence of an electric field is investigated. A high speed camera is used to capture the footage and finite element method (FEM) is used to simulate the multi-physics phenomenon on two polymeric surfaces, namely, silicon rubber (SiR) and PTFE (polytetrafluoroethylene). A ‘creepage’ motion was observed. The inception of motion and the movement speed are analysed in correlation with various surface conditions. Models are established to estimate the moisture and potential discharge characteristics on the inclined polymeric surfaces. They are further utilized to analyse the actual insulators subject to wet conditions.

## 1. Introduction

The distortion and movement of rain drops in an electric field has been the subject of a number of experimental research studies. High voltage AC outdoor insulators can be distinct by their bulk insulation material as ceramic insulators (glass or porcelain) or composite insulators (polymeric or non-ceramic insulators). Composite insulators consist of pultruded glass cores and polymeric shed sheaths are fast becoming the main choice of materials because of their improved wetting behaviour and greater degree of hydrophobicity [1,2]. The discharge activity on the surface of polymeric insulator surfaces is one of the ageing mechanisms leading to insulator failure. Such activity between the rain droplets causes the production of radicals that chemically react with the surface of the insulator, thereby altering the properties of the insulator material. The practical aspect of the subject seems to be an important factor in justifying the extensive work carried out on electrical discharges from rain drops on polymeric surfaces.

The surface of operational polymeric insulator experiences hydrophobicity weakness and even loss due to the surface discharge of the rain droplet. Due to the reduced hydrophobicity, the rain droplets coalesce and form ‘rivulets’ of conductivity, which bridge the insulation path on the insulator surface leading to the flashover between HV and ground. Most experimentation revolves around discharges between rain drops on the top of flat horizontal surfaces. As the weather-proof sheds of insulators are neither horizontal nor planar, it is worth studying the behaviour of rain droplets in more complex situations.

This article aims to investigate the behaviour of a rain droplet on an inclined insulator surface under the influence of intense AC electric field. This unique experiment and its results are necessary to further understand the behaviour of moisture [3] and ageing process for polymeric surfaces, especially their application within power systems for insulators and bushings.

## 2. Literature Review

The study of thunderstorms inspired the early work on the impact of electrical fields on rain drops and much of the activity was concerned with the distortion and break-up of individual drops in high fields. The discharges on the surface of polymeric insulators are important in the ageing mechanisms of insulators. Since the introduction of hydrophobic polymeric insulators, a considerable amount of work has been conducted addressing the electrical discharges from rain droplets on polymeric surfaces. Rowland [4] highlighted the critical role played by the local ageing of the polymers’ surface in the formation of ‘wet fingers’ of conductivity. Karady et al., for example, describes the formation of the filaments between rain droplets on aged commercial silicon rubber insulators and a mechanism by which this led to flashover of single sheds [5,6,7].

The vibration and distortion of rain drops in the presence of an alternating field (ac) is well established [8,9,10,11]. A rain droplet in a strong electric field deforms as a result of the interaction of electrostatic force and the surface tension of the rain droplet [12]. The movement of rain droplet in alternating field (ac) is characterized by periodic vibration or fluctuation. Higashiyama and Yamada investigated in detail the behaviour of a rain droplet placed on the surface of the hydrophobic polymeric sheet in presence of an ac field. They demonstrated the change in droplet shape during vibration, showing that the droplet is deformed and synchronized with the ac field [8]. Their study revealed that the frequency and the volume of rain droplet greatly affect the amplitude of vibration.

An electric field alone can force raindrops up an incline in order to move away from a high-stress region pointing out the importance of gravity on inclined surfaces [13]. Phillips et al. and Cheng et al. have shown elongation of single raindrops [14,15]. They observed that raindrops do not change their shape gradually, but in a series of steps, and do not necessarily recover their shape after the field is removed. Krivda and Birtwhistle [13] explained that natural vibrations of a rain drop change its shape during the ac cycle and hence can reduce the insulation path. This increases the risk of flashover. It was shown that when several raindrops coalesce they can bridge a significant distance of insulation and hence discharges appear between them. The corona discharge takes place at the triple junction area of the rain–air–solid interface as the electric field at this point is intensified due to the difference in permittivity of silicon rubber, air and the rain droplet [16].

The corona discharge destroys the hydrophobicity of the insulator and thus a long chain of elongated drops is formed. Under the influence of electric field, this can lead to the flow of currents over the surface of the insulation [5].

The discharge activity between rain droplets on the insulator surface plays an important role in its ageing mechanisms. It is also critical in the processes leading up to the flashover of these insulators in highly polluted or marine environments. However, limited research has been conducted on the impact of surface shapes and dimensions on surface hydrophobicity and water sliding behaviour.

## 3. Methodology

To address this gap in the literature, this article aimsu to design a specific experimental set up to investigate the factors affecting the rain droplets’ dynamic movement on an inclined insulation surface. To simplify, a flat inclined surface was used with the capability to vary its inclined angle. The first step was to investigate the behaviour of the rain droplets on an inclined plane in the absence of electric field [17]. This will facilitate the design process while identifying the key variables that need to be controlled. In the second step, the experiments were conducted in a high voltage environment. The aims and objectives of the study can change depending upon need and setup availability/feasibility.

### 3.1. In the Absence of an Electric Field

**Figure 1:**
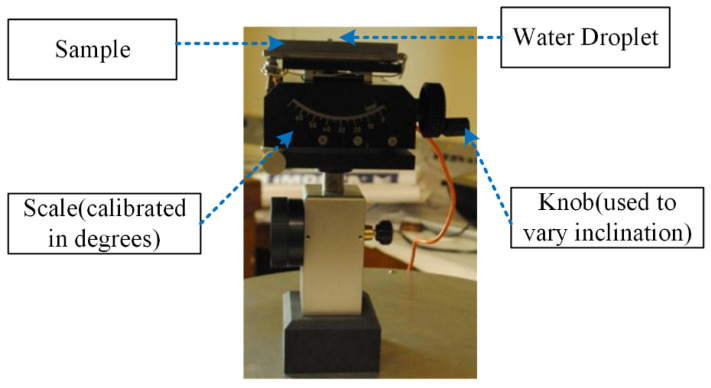
Test up to measure inclination angle.

Silicon rubbers with different additives are materials of high interest for high voltage composite insulators. A number of other materials were also chosen for investigation in order to examine the role of surface energy or hydrophobicity. These materials included glass, PTFE and a commercial silicon rubber-based material (SiR). A drop of de-ionised water was placed on the sample surface by a pipette and the angle of inclination gradually increased until the droplet started to move. The experiment was repeated with three different sizes of droplet (50, 90 and 140 μL) and material types (SiR, PTFE and Glass). The diagram of the test setup is shown in Figure 1.

**Figure 2:**
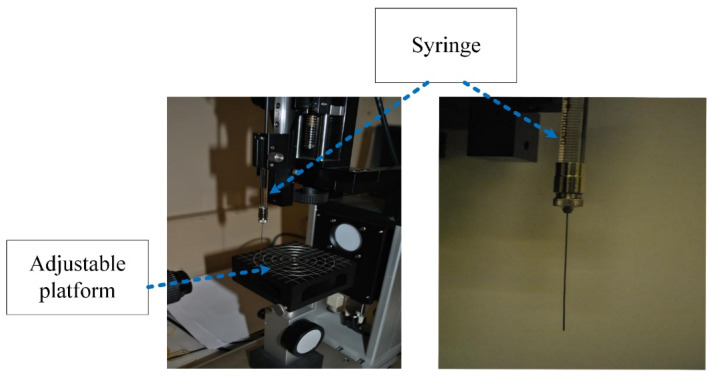
Syringe unit of the optical contact angle measuring device (Data Physics OCA Series).

The hydrophobicity of each material was determined by measuring the contact angle with a 100 μL drop of de-ionised water. The geometry of the point of contact between the liquid, solid and air is the key to controlling the process, so this is a particularly useful measurement in that context. Figure 2 shows the diagram of the syringe unit of the optical contact angle measuring device.

### 3.2. In the Presence of an Electric Field

**Figure 3:**
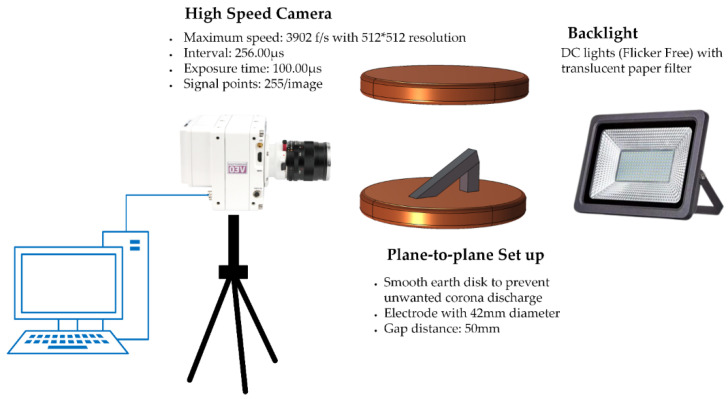
Diagrammatic overview of the experimental setup.

The basic understanding developed through investigating rain droplet behaviour on different materials (without E-field) helped to design the experiment. The experimental setup consists of two large cylindrical metal electrodes with rounded edges. The electrode surfaces were smooth with little or no asperities in order to obtain uniform electric field. A silicon rubber sample with an inclination of 30 was used as a test material. The metal electrodes were separated by about 50 mm. A 50 μL drop of de-ionised water was placed in the central region on top of the insulator sample. The droplet size (volume) was controlled by the electronic syringe unit of the optical contact angle measuring device. A high speed camera (imaging rate 3902 fps) was used to observe and record the movement of the rain droplet under the influence of high AC electric field. A diagram of the experimental setup is shown in Figure 3.

**Figure 4:**
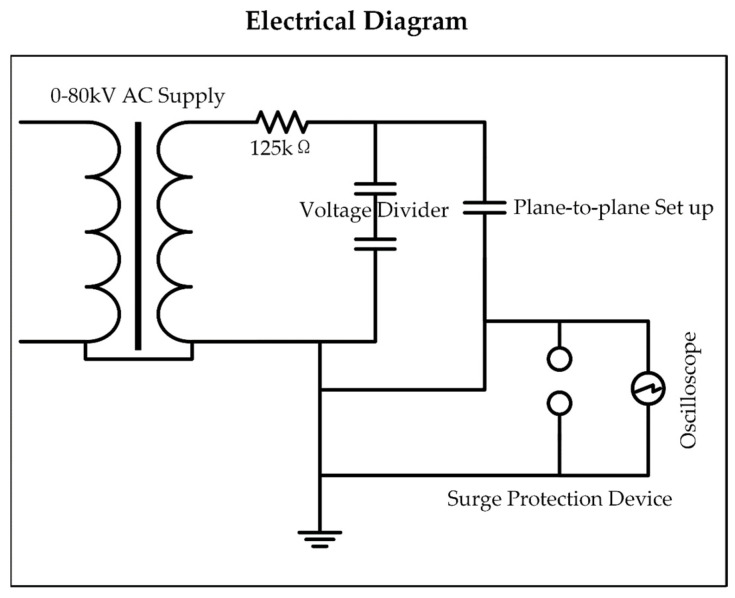
Diagram of the test circuit.

The electrical diagram of the same experiment is shown in Figure 4. The components include a high voltage transformer, the rated voltage of which is 80 kV AC (frequency 50 Hz), and a 125 kΩ current limiting resistor. The circuit also contains a surge protection device and a voltage divider. A digital storage oscilloscope was used to monitor the voltage waveforms.

A ‘break down test’ was conducted in order to find the maximum voltage that can be safely applied to the test setup before the dielectric (air between electrodes) breaks down. A fairly large rain droplet with the volume 100 μL was applied to the sample surface and the voltage was gradually increased. The breakdown occurred at 55 kV and hence it was decided to perform all experimentation below the 35 kV voltage level.

**Figure 5:**
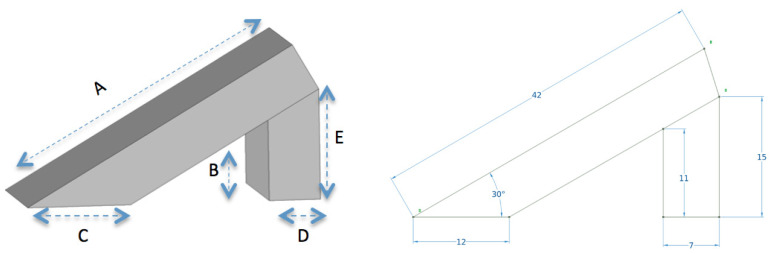
Silicon rubber sample with 30 degrees inclination.

The silicon rubber used in the experiment was designed using basic geometry. The thickness of the material was 7 mm. A 30° inclined silicon rubber sample was made using a sharp cutting tool as shown in Figure 5. The dimensions of the sample are shown in Table 1.

### 3.3. Numerical Simulation Using the Finite Element Method (FEM)

When the rain droplet is under the electric field, the electric field force drives the movement and deformation of the rain droplet, which leads to the distortion of the field strength and changes the size of the electric field force [18,19]. The interaction between the two is a coupling problem of electricity and fluid dynamics, which is defined as electrohydrodynamics. After a sensitivity study of various simplified versions of the simulation in comparison with the measurements, the FEM was identified as the most effective tool to understand the physical mechanism behind the droplets’ dynamics.

## 4. Results and Discussion

### 4.1. Contact Angles and Inclination Angle of Samples

The experimentation conducted in absence of electric field was quite useful in developing a basic understanding about key concepts, such as surface hydrophobicity and water contact angles. The contact angles between rain drops on different surfaces are shown in Table 2; each value given is an average of three measurements.

Table 3 shows the inclination angle at run-off for different materials and droplet sizes (volume). In the experiments carried out, a fresh sample of material was used for all readings. The line of sight was kept perpendicular to the scale while taking the readings in order to avoid a parallax error.

The results in Table 2 show that the highest contact angles were seen for silicon rubber sample. This can be explained by the excellent hydrophobicity of silicon rubber. Water molecules were seen to form discrete droplets on the surface of the insulation when the material is highly hydrophobic.

Table 3 shows that, for a given volume, a greater degree of inclination was required to run off a water droplet from the SiR surface compared to PTFE and glass. The surface properties of these materials, such as surface roughness and hydrophobicity, are important in water droplet movement. The results were important in the design of the experiment conducted in presence of high electric field (AC). It provided the range of the droplet sizes and materials that can be used to meet the research specifications.

### 4.2. Creepage Phenomenon on the Insulator Surface

The main highlight of the experiment was the identification of a ‘Creepage Phenomenon’, which has not been observed earlier in the literature. The vibration and distortion of water drops on horizontal surfaces under the influence of an alternating field (AC) is well established. It was observed that, if the water droplet placed on an inclined insulator surface is subjected to an ac field, it tends to creep along the surface. Additionally, the movement is affected by numbers of factors, including the strength of field, volume of water droplet, type of the insulating material and the roughness of insulator surface. This dynamic behaviour of water droplet can play an important role in the ageing mechanisms of composite systems.

**Figure 6:**
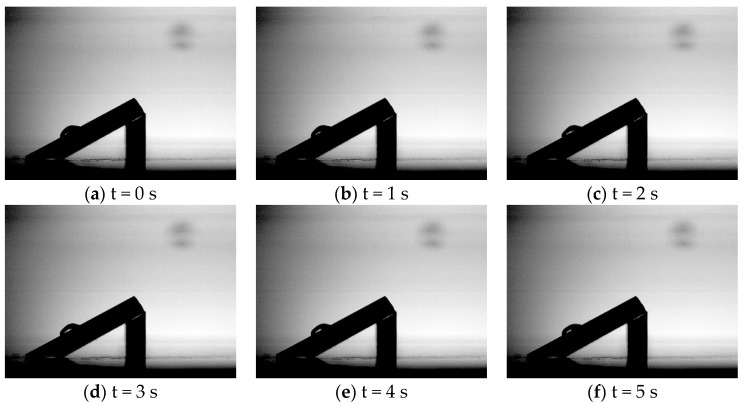

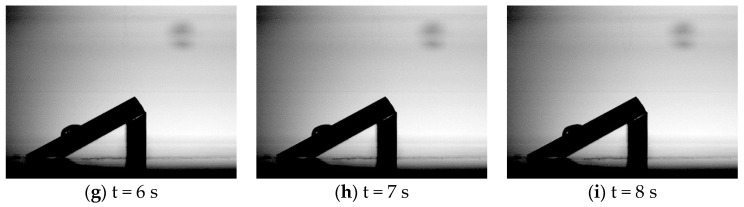
Still pictures from high-speed camera footage.

The voltage was uniformly increased from zero kV in steps of 5 kV. The ‘Creepage Phenomenon’ was seen at 30 kV (AC) using the experimental setup shown in Figure 3. The high-speed camera captured the movement and distortion of the water droplet placed on the inclined surface, which is shown in Figure 6. It can be clearly seen that the droplet creeps down the insulator surface during the course of the experiment.

Figure 6 shows the movement of the water droplets corresponding to footage of 8 s. It was found that:

The water droplet in strong electric field deforms as a result of the interaction of electrostatic force and the surface tension of the water droplet. Additionally, it vibrates with many vibration modes throughout the creepage movement. The deformation and vibration modes of the water droplet depend on a number of factors. The factors include the magnitude of the applied field, the surface tension, droplet size (volume) and the water density of the water droplet [13,20,21]. Additionally, the actual insulator surface has a non-uniform electric field distribution. This means that it is possible to find many vibration modes occurring simultaneously on its surface.

Upon careful observation of the high-speed camera footage, the water droplet was seen to elongate in the action of electric field. The change of water droplet shape would have a significant influence on the insulation performance of insulators.

### 4.3. Speed of Creeping Movement

**Figure 7:**
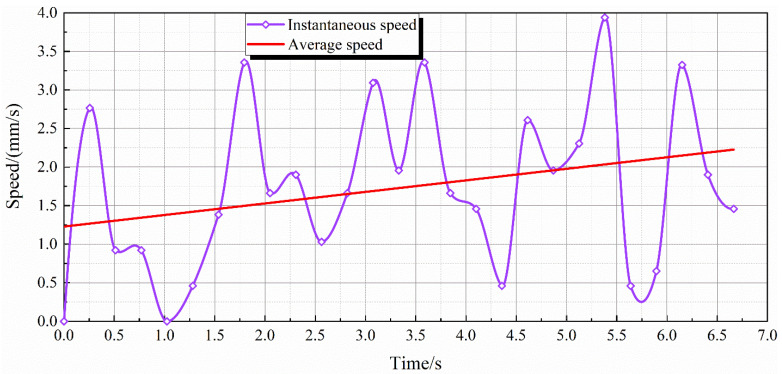
Speed of rain droplet movement with time.

The speed of the water droplet creeping down the insulator surface was calculated using MATLAB. This helped to quantify the experiment. The relationship between time and the speed of movement is shown in Figure 7. The graph expresses the variation of instantaneous speed against time. The average speed was also calculated, which is seen to increase with time.

Figure 7 shows that:

The maximum instantaneous speed is recorded as 3.94 mm/s at 5.38 s. The variation in instantaneous speed can be caused due to a number of factors, especially hydrophobicity and surface roughness.

Overall, the average speed of water droplet motion increases with time. The reason for this is that the combined forces on the water droplet are the driving forces.

It was seen that the water droplet placed on the SiR sample under the influence of ac electric field vibrates. This results in the deformation of the water droplet shape. The change in shape can cause the enhancement of a local electric field in the triple junction area of water–air–solid. Cheng [15] points out that the surface of composite insulators can experience hydrophobicity loss as a result of local electric field enhancement. Corona discharge may appear at the triple junction as the electric field is most intensified in that area [22]. This can facilitate the movement of the water droplet on the sample surface and thereby an increase in instantaneous speed is observed.

The energy transfer mechanism can also play an important role in justifying the speed variation. The energy (surface tension) of vibrating water droplet is transferred to the kinetic energy of the moving droplet and vice versa. After each subsequent movement, the dynamics change, resulting in different conditions needed to initiate the movement again.

### 4.4. The Occurance of the Creeping Phenomenon

It was quite interesting to observe that the phenomenon did not recur, despite many attempts to reproduce it. Both old and new silicon rubber samples were employed to see the reproducibility of the experiment. Later, the angle of inclination was also increased to 40 degrees to see the effect of level of inclination on water droplet movement. This had no impact on the water droplet movement as the creepage phenomenon was not observed at all. An increase in the electric field resulted only in severe vibration and distortion of the water droplet, but no movement. The same experiment was repeated on PTFE (another hydrophobic insulator) in order to examine the effect of surface energy or hydrophobicity. This experiment again did not see any creeping of the water droplet subject to same experimental conditions.

The last stage involved changing the droplet size (volume) to investigate its effect on the water droplet movement. In this case, the ‘Creepage Phenomenon’ was successfully repeated four times with a water droplet of about 75 μL. This indicates two main possibilities: the water droplet size measured as 50 μL in the initial experiment was inaccurately dispensed by the electronic syringe system, or there are some other unrevealed factors governing the movement of water droplets on inclined surfaces.

### 4.5. Electric Field with an Insulator

**Figure 8:**
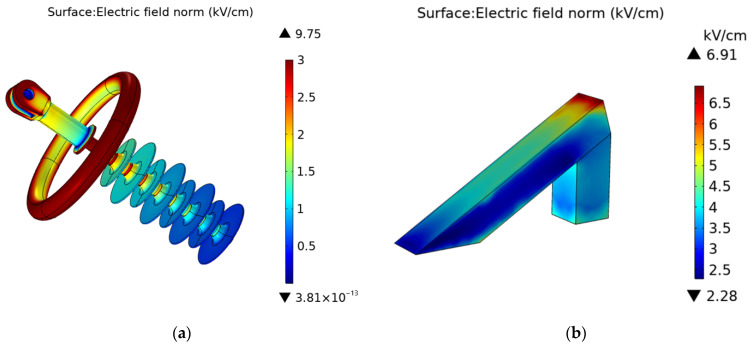
(**a**) Electric field distribution on a real insulator; (**b**) Electric field distribution on a sample (SiR).

The experimental set up was designed in a way to simulate the electric stress of real insulators in service. This was quite important in developing a useful experiment whose relevance can be directly related to the practical world of overhead line composite insulators. The finite element method was employed as an effective tool to compute the electric field. Figure 8a shows the electric field distribution of an insulator in service. It can be seen that the electric field distribution on a real insulator varies from 0 to 9.754 kV/cm along the insulator length.

The electric field distribution on the silicon rubber sample surface is shown in Figure 8b. The top electrode is connected to the high voltage end, so the electric field intensity increases in magnitude from bottom to top on the sample surface. The graph shown in Figure 8 confirms that the electric field on the sample surface simulates the electric stress experienced by real insulators. This shows that a great proportion of sample surface experiences the same electric stress as that of real high voltage composite system insulator.

## 5. Conclusions

It can be concluded that the water droplet placed on an inclined insulator surface vibrates with various modes. This is due to the reason that the insulators in service experience different electric field distribution on its surface and form wide range of droplet sizes. A ‘Creepage Phenomenon’ was observed where the water droplet (placed on an inclined polymeric surface) tends to creep along its surface in presence of high AC electric field. The experiment proved that this phenomenon is water-droplet-size dependent. A water droplet of about 75 µL is needed to observe the creepage movement of the water droplet on a 30 degrees inclined silicon rubber sample. The average speed of movement is seen to increase with time. The effect of hydrophobicity (for fixed volumes) did not have an important impact on the creepage movement. The outcomes from the indicated that this new phenomenon, so called ‘creepage’, plays a significant role in the ageing process of high voltage composite insulators.

## Figures and Tables

**Table 1:** Sample dimensions.

A	B	C	D	E
42 mm	11 mm	12 mm	7 mm	15 mm

**Table 2:** Contact angles between water droplets on different materials.

Materials	SiR	PTFE	Glass
Contact Angles (°)	108	100	38

**Table 3:** Inclination angle at run-off.

	Volume	50 μL	90 μL	140 μL
Sample Type				
SiR		52°	40°	32°
PTFE		49°	37°	28°
Glass		33°	21°	16°

## Data Availability

Data sharing not applicable.
